# Freestanding three-dimensional core–shell nanoarrays for lithium-ion battery anodes

**DOI:** 10.1038/ncomms11774

**Published:** 2016-06-03

**Authors:** Guoqiang Tan, Feng Wu, Yifei Yuan, Renjie Chen, Teng Zhao, Ying Yao, Ji Qian, Jianrui Liu, Yusheng Ye, Reza Shahbazian-Yassar, Jun Lu, Khalil Amine

**Affiliations:** 1 School of Materials Science and Engineering, Beijing Institute of Technology, Beijing Key Laboratory of Environmental Science and Engineering, Beijing 100081, China; 2 Chemical Sciences and Engineering Division, Argonne National Laboratory, 9700S. Cass Avenue, Lemont, Illinois 60439, USA; 3 Collaborative Innovation Center of Electric Vehicles in Beijing, Beijing 100081, China; 4 Department of Mechanical and Industrial Engineering, University of Illinois at Chicago, Chicago, Illinois 60607, USA

## Abstract

Structural degradation and low conductivity of transition-metal oxides lead to severe capacity fading in lithium-ion batteries. Recent efforts to solve this issue have mainly focused on using nanocomposites or hybrids by integrating nanosized metal oxides with conducting additives. Here we design specific hierarchical structures and demonstrate their use in flexible, large-area anode assemblies. Fabrication of these anodes is achieved via oxidative growth of copper oxide nanowires onto copper substrates followed by radio-frequency sputtering of carbon-nitride films, forming freestanding three-dimensional arrays with core–shell nano-architecture. Cable-like copper oxide/carbon-nitride core–shell nanostructures accommodate the volume change during lithiation−delithiation processes, the three-dimensional arrays provide abundant electroactive zones and electron/ion transport paths, and the monolithic sandwich-type configuration without additional binders or conductive agents improves energy/power densities of the whole electrode.

## Abstract (web summary)

Degradation and low conductivity of transition metal oxide anodes cause capacity fading in lithium ion batteries. Here the authors make freestanding 3D copper oxide/carbon nitride core-shell nanoarrays which accommodate volume change, provide electro-active zones and facilitate rapid charge transport.

## Graphical abstract

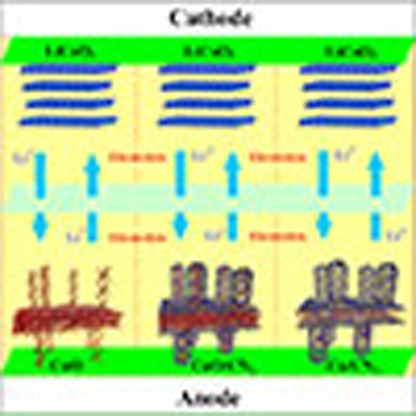

Stringent requirements for reliable, fast and highly efficient energy technologies are ever increasing because of the growing electronics market and electric vehicle industry. Currently, lithium-ion batteries dominate as power sources for portable electronics and are regarded as the most promising power sources for electric vehicles and grid applications. However, these widespread and long-term applications still require better batteries in terms of performance, safety and cost, which can be achieved by better utilization of electrode materials and/or an optimized design of battery configurations^1^,^2^,^3^,^4^.

Even though widely used, current commercial lithium-ion batteries containing a graphite anode have limited specific capacity and energy density in both portable and transportation applications^5^. Thus, new, low cost, safe anode materials with higher cycling capacity and power capability are urgently needed. In past decades, considerable efforts have been devoted to searching for superior anode materials, such as lithiated carbons, Si/Sn-based materials, Ti-based oxides and other metal oxides/sulfides/nitrides/fluorides/phosphides^6^,^7^,^8^,^9^,^10^,^11^,^12^,^13^,^14^,^15^. The 3d transition-metal oxides (*M*_*x*_O_*y*_, *M*=Co, Ni, Fe, Mn, Cu and so on) have been proposed as promising anodes because they are capable of incorporating more than one Li per 3d metal, giving significantly higher electrochemical capacities than those of conventional graphite anodes. The mechanism of Li reactivity of metal oxides differs from the classic Li-intercalation or Li-alloying processes^16^. The metal oxides are reduced to metal nanoparticles through a conversion reaction that involves the formation of Li_2_O:


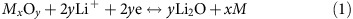


Similar to the Li-alloying process, the conversion reaction leads to volume variation on electrochemical cycling. Therefore, pure metal oxide electrodes typically suffer from poor cyclability and poor power capability. To solve these issues, researchers have tried various strategies such as reducing particle size or using nanocomposites^17^,^18^. Reducing particle size is considered as an effective approach to reduce volume variation. Indeed, many nanostructures based on 3d metal oxides, such as nanospheres, nanowires, nanorods, nanotubes, nanoplates, and hollow structures, have been summarized in a wealth of published articles^10^,^19^,^20^,^21^,^22^. Using nanocomposites or hybrids by integrating metal oxides with conducting additives, such as graphitized carbons, carbon nanotubes, graphenes, conductive polymers and so on, have also been reported to improve the utilization of the active materials^23^,^24^,^25^,^26^. However, these traditional strategies appear far from practical applications due to limited cycling performance and/or complex fabrication process.

Among metal oxides, copper oxide (CuO) has received attention owing to its advantages of high theoretical capacity (670 mAh g^−1^), low cost, facile synthesis and environmental benignity^26^,^27^,^28^,^29^. Good cycling stability and high-rate performance are two critical factors in the development of metal oxide anodes. Herein, we induce a monolithic synthetic technique^30^,^31^, directly growing active materials onto metallic current collectors, to maintain high conductivity while avoiding the use of binders and conductive agents. Here, we rationally design a freestanding, three-dimensional (3D) sandwich-type composite electrode based on CuO/carbon-nitride (CN_*x*_) core–shell nano-architecture. The electrode is prepared via thermal oxidization combined with radio-frequency magnetron sputtering. The typical synthesis route and deposition mechanism are illustrated in Fig. 1. Nanowire arrays of CuO are obtained through the oxidation of copper substrates (foil, grid and net), followed by deposition of ultrathin CN_*x*_ films through radio-frequency sputtering of a graphite target in a high-purity N_2_ gas, forming 3D CuO/CN_*x*_ core–shell nano-architectures. CN_*x*_ films are deposited onto both sides of the CuO nanosubstrate, forming a sandwich-type CN_*x*_/CuO/CN_*x*_ composite. In this electrode, CuO/CN_*x*_ core–shell nanocables serve as active materials; the CN_*x*_ coating provides good electron conduction and flexibility, as well as mechanical strength; and the 3D arrays have large surface areas, good permeability and abundant active sites. Furthermore, the 3D nanocomposites can be reduced into Cu/CN_*x*_ core–shell nanocable arrays. These different 3D nanosubstrates (CuO, CuO/CN_*x*_ and Cu/CN_*x*_) are fabricated as electrodes and show stable cycling in coin-type cells for over 200 cycles. Hereafter, we refer to these specific composites with the acronyms CNE (CuO nanowire electrode), CCNE (CuO/CN_*x*_ nanocable electrode) and CNNE (Cu/CN_*x*_ nanocable electrode).

## Results

### Physical characteristics

**Figure 2:**
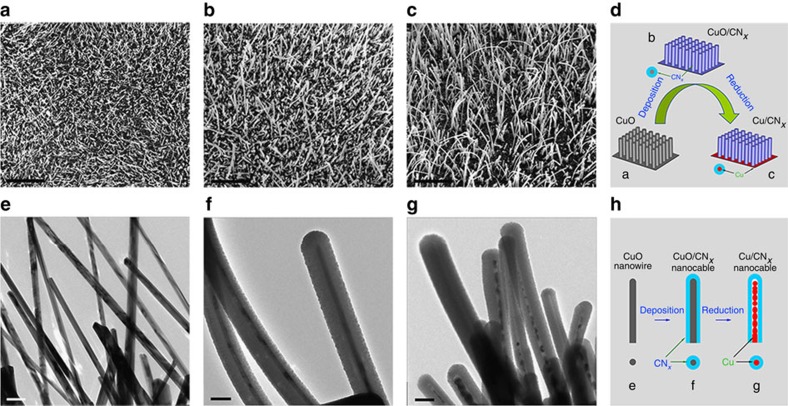
Structure and morphology of nanocomposite electrodes. s.e.m. and TEM micrographs of the CNE (**a**,**e**), CCNE (**b**,**f**) and CNNE (**c**,**g**) based on the copper foils. (**d**,**h**) Schematic of the oxidation, deposition and reduction processes of the nanosubstrates. Scale bars in **a**–**c**, 20 μm; scale bars in **e**–**g**, 200 nm.

The 3D nanoscale morphologies of CNE, CCNE and CNNE grown on copper foil are observed by s.e.m. and transmission electron microscopy (TEM). In Fig. 2a–e, it is obvious that a high density of uniform CuO nanowires, with diameters in the range of 50−100 nm and lengths of 10−20 um, grows vertically onto the substrate. Similarly, in Fig. 2a,b dense array of uniform CuO/CN_*x*_ nanocables is observed on the substrate. The thickness of the CN_*x*_ film after 40 min sputtering is ∼140 nm, with a deposition rate of 3.5 nm min^−1^, as shown in Fig. 2f. After the reduction, in Fig. 2c, the entire surface of the substrate is still covered by a high density of Cu/CN_*x*_ nanocables, which appear to be thinner and more curved than the CuO/CN_*x*_ nanocables. The shape change is mainly caused by the volume decrease that occurred during the reduction of wire-like CuO cores into copper nanoparticles, forming new hollow Cu/CN_*x*_ nanocables, as shown in Fig. 2g. The entire process of oxidation, deposition and reduction of these nanosubstrates is illustrated in Fig. 2d–h.

**Figure 3:**
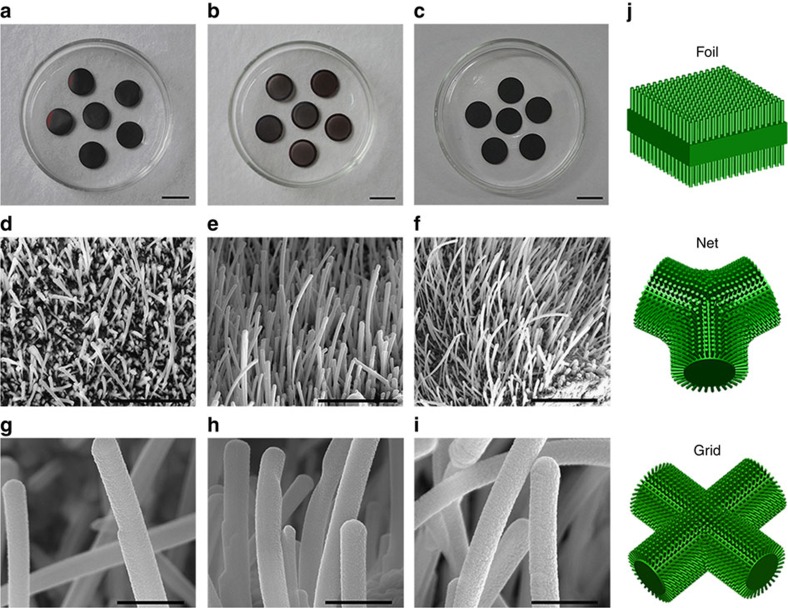
Structure and morphology of the CCNEs based on various substrates. Digital images and corresponding s.e.m. micrographs of the CCNEs based on the foil (**a**,**d**,**g**), net (**b**,**e**,**h**) and grid (**c**,**f**,**i**) substrates, and corresponding 3D nano-architectural models (**j**). Scale bars in **a**–**c**, 1 cm; scale bars in **d**–**f**, 10 um; scale bars in **g**–**i**, 1 um.

The morphologies of CNEs (Supplementary Fig. 1) and CCNEs (Fig. 3) prepared using different copper substrates are also observed by s.e.m. Figure 3a–c are the optical photos of CCNE films with diameters of 1.0 cm based on foil, net and grid substrates, respectively. The surfaces of the copper substrates are tarnished after heat treatment in O_2_ at 600 °C followed by graphite deposition in N_2_ for 40 min. Figure 3d–f show the corresponding s.e.m. images, which display the 3D nano-architectures of high-density wire-like nanostructures over the entire surfaces of these substrates. Among these substrates, the nanostructures grown on the inner edges of the nets and ridges of the grids appear to be much denser and longer than those grown on the surface of the foils. Figure 3g–i exhibit high-magnification s.e.m. images, which confirm a uniform diameter of 300−400 nm for these wire-like nanostructures. Figure 3j illustrates the 3D nano-architectural models of these CCNEs.

**Figure 4:**
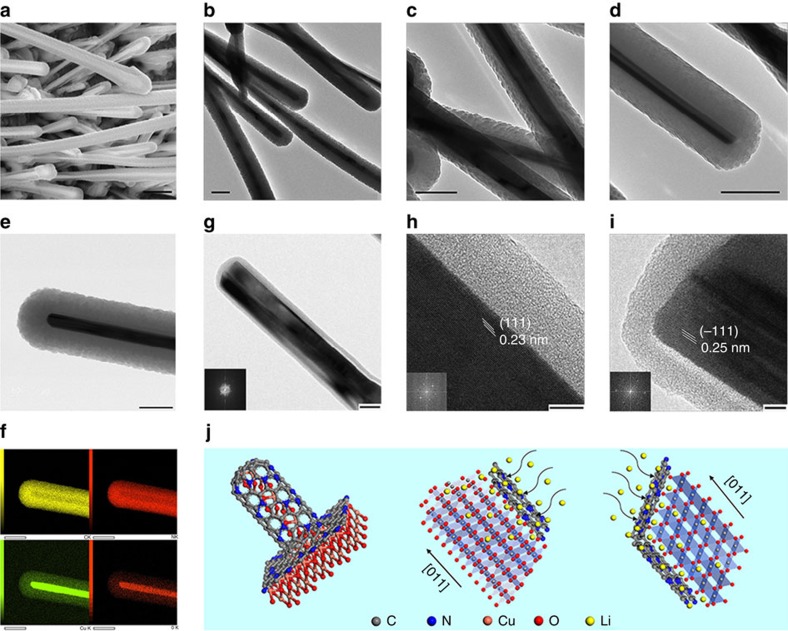
Core–shell structure of the CCNEs. s.e.m. (**a**) and TEM (**b**) micrographs of the CCNE after 30 min deposition. TEM micrographs of the CCNEs after 20 min (**c**) and 40 min (**d**) deposition. STEM image (**e**) and EDX elemental mapping (**f**) of the core–shell nanocable. High-resolution TEM images (**g**–**i**) of the nanoscale after 5 min deposition, and corresponding micro-mechanism models (**j**). Scale bar in **a**, 1 um; scale bars in **b**–**f**, 200 nm; scale bar in **g**, 40 nm; scale bars in **h** and **i**, 5 nm.

The core–shell nanostructures in CCNEs are investigated by TEM. It can be observed from the s.e.m. image (Fig. 4a) that the nanostructure array exhibits clear core–shell structures. Therefore, the wire-like nanostructure is considered to be a nanocable. In Fig. 4b–g, TEM images reveal that the nanocables have inner cores with diameters of 50−100 nm, and outer shells with diameters of 15−140 nm. In general, the diameter of the CuO cores is determined by the oxidation temperature and time, and the thickness of the CN_*x*_ shells is mainly controlled by adjusting the deposition time. The element distribution in the nanocables is analysed by energy dispersive X-ray (EDX) spectroscopy. In Fig. 4f, the EDX elemental mapping of the nanocable (Fig. 4e) shows an intense signal for Cu and O in the middle and a uniform distribution of C and N across the shell, suggesting a uniform coverage of the CN_*x*_ film over the CuO core. The crystal structure of the nanocables is observed in the high-resolution TEM of Fig. 4g. It is obvious that a wire-like CuO core is covered by a nanosized microporous carbon-nitride shell, and the middle of the CuO core is clearly divided by a (111) twin plane along the longitudinal axis, which has been reported in CuO nanowires synthesized via heating copper substrates in air by Jiang *et al*.^32^. In Fig. 4h,i, note that each side of the CuO core is, indeed, a single crystal with well-defined fringe space pattern. The interplanar spacing for each side is 2.32 and 2.52 Å, which correspond well to the (111) and (−111) spacings in monoclinic CuO, respectively^32^,^33^,^34^. This finding is also in good agreement with the first-principle calculations that (111) and (−111) planes have the lowest surface energies under ambient conditions and, hence, should be the most preferential facets of CuO nanowires^35^.

The structure and chemical composition of all samples are further characterized by X-ray diffraction (XRD), Raman spectroscopy, Fourier transform infrared spectroscopy (FT-IR) and X-ray photoelectron spectroscopy (XPS), as shown in Supplementary Fig. 2. The XRD results indicate that the obtained copper oxides nanowires have a monoclinic structure, and a weak peak at 26.2° corresponds to the (002) plane of graphite-like carbon nitrides. The Raman results also confirm the single-phase property and high crystallinity of CuO nanowires, while in the CCNE and CNNE samples, two intense bands at 1355 and 1593, cm^−1^ are attributed to the D-band (disorder-induced phonon mode) and G-band (graphite band) of carbon nitrides, respectively^36^. It is generally accepted that the intensity ratio I_D_/I_G_ can be used to estimate the degree of disorder in carbon materials^37^. Note that the I_D_/I_G_ ratio of the CNNE is higher than that of the CCNE, indicating that the thermal reduction process generates extrinsic defects in the carbon-nitride framework. The FT-IR analysis reveals the oxidation of Cu^2+^ in the CuO nanowires and the graphitic structure of the CN_*x*_ film. The XPS analysis provides further evidence for the significant chemical nature of nitrogen incorporation in the carbon matrix. In the N spectrum, the peak shows an asymmetry, where the major component at 398.7 eV is due to the pyridinic nitrogen, and shoulder peaks at 400.1−401.2 eV are assigned to the pyrrolic nitrogen in the terminal C−N groups and quaternary nitrogen in the graphite C−N bonds^36^,^38^. This finding indicates that the nitrogen-substituted carbon creates more pyridine- and pyrrole-like defects in the carbon framework and generates more active sites for the Li storage. Due to the higher electronegativity of nitrogen (3.5) than carbon (3.0), and its smaller diameter, the stronger interaction between the carbon nitrides and Li also facilitates the penetration of Li across the defects^36^,^37^,^39^. Further details on chemical and structural characterization can be found in Supplementary Note 1.

### Electrochemical properties

**Figure 5:**
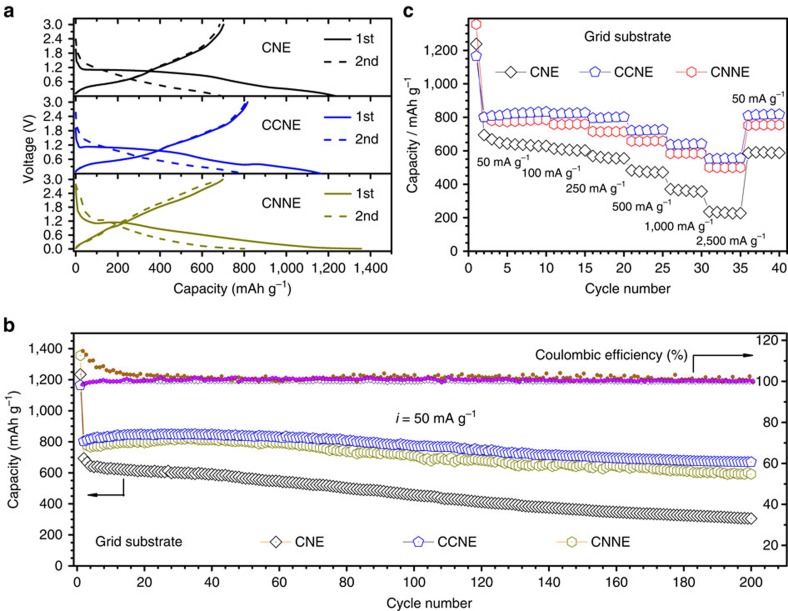
Electrochemical characterization of nanocomposite anodes in half-cells. Voltage profiles (**a**), cycle performance (**b**) and rate performance (**c**) for CNE, CCNE and CNNE samples based on the grid substrate. (In this and subsequent figures, all specific capacities of CCNEs are based on the total mass of the active materials contain CuO and CN_*x*_ in the core–shell structure).

The battery performance of our hierarchical structured electrode is evaluated by galvanostatic charge–discharge cycling of the 2025 coin-type cells. Half-cells with a metallic Li counter electrode are used to evaluate electrochemical properties of these nanocomposite electrodes. In Fig. 5a, the cells are run at a current density of 50 mA g^−1^; the CNE, CCNE, and CNNE based on grid substrates deliver an initial discharge capacity of 1234.8, 1164.8 and 1355.6 mAh g^−1^, respectively. In the next cycle, the reversible discharge capacity of the CNE, CCNE and CNNE is 692.9, 803.6 and 801.8 mAh g^−1^, respectively, with capacity retention of 56.2, 69.0 and 59.1%, respectively. These results suggest that the CuO core and CN_*x*_ shell in the nanocables both provide deintercalation capacity, and that the CCNE with core–shell architecture exhibits the highest reversible capacity and capacity retention. The extra capacity above the theoretical value of CuO (670 mAh g^−1^) is believed to be due to the reductive decomposition of the electrolyte and subsequent formation of solid-electrolyte interphase (SEI) layers, which are inferred from cyclic voltammetry (Supplementary Fig. 3 and Supplementary Note 2). The SEI layers covering the surface of active nanocomposites prevent further reaction between the components of the electrolyte and the active materials^40^. However, note that there is a large initial capacity loss, especially for the CNE and CNNE, which is due to the high surface areas of nanowire arrays. Figure 5b shows the cycle performance over 200 cycles of different nanocomposite electrodes based on the grid substrate. From the 2nd to the 200th cycle, their capacity retention is 44.0, 83.3 and 73.8%, respectively. After 200 cycles, the CCNE still delivers a high capacity of 669.0 mAh g^−1^, which is almost twice the theoretical capacity of graphite and also higher than that of the CNE (305.1 mAh g^−1^) and CNNE (592.1 mAh g^−1^). Also note that the coulombic efficiency of the CCNE rapidly increases to ca. 99% after the first 5 cycles, and remains stable throughout the cycles, indicating that the SEI layers remain intact, allowing reversible cycling after the surface reactions are completed. This result suggests that the CCNE shows the highest reversible capacity and the best cycle performance, indicating electrochemical reversibility and structural stability. Figure 5c shows rate performance versus cycle life. In general, the capacity retention of all electrodes decreases with increasing current density from 50 to 2500, mA g^−1^. Compared with the CNE, the CCNE and CNNE show better stability on cycling at each current rate, and almost recover their original capacity values when the current density is restored to 50 mA g^−1^. At the current density of 2,500 mA g^−1^, the CNE, CCNE and CNNE deliver an average discharge capacity of 230.5, 553.7 and 500.3 mAh g^−1^, respectively. The rate performance exhibited, particularly by CCNE, is excellent for a metal oxide anode. This finding reveals that the CCNE shows good charge transfer kinetics and stable structural integrity. The reductive CNNE also exhibits good electrochemical performance as a promising carbon-nitride candidate for anode materials. It is likely that the high N doping in the CN_*x*_ layer (∼20 at%, in this work) creates abundant defects that are well known to increase the capacity of the engineered carbon for their fast reaction with lithium^37^. Moreover, the N doping can also enhance the electronic conductivity, which additionally contributes to the exceptional rate performance^38^. However, initial coulomic efficiency and cyclability is poorer in CNNE than in CCNE, mainly due to the morphological/structural changes during the thermal reduction, which yield higher surface areas and reduce the structural stability of the CNNE (Supplementary Fig. 4).

**Figure 6:**
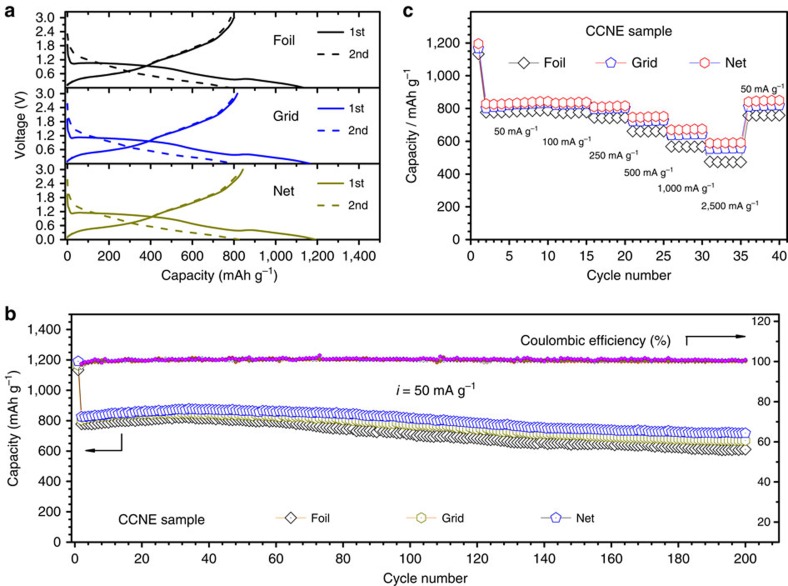
Electrochemical characterization of CCNEs in half-cells. Voltage profiles (**a**), cycle performance (**b**) and rate performance (**c**) for CCNEs based on the foil, grid and net substrates.

We investigated the effect of the different substrates on the cell performance. Figure 6a,b show a set of parallel charge–discharge profiles and capacity retention over 200 cycles for comparison, respectively. The initial discharge capacity of the CCNE based on the foil, grid and net is 1134.1, 1164.8 and 1190.6 mAh g^−1^, respectively. After 200 cycles, their remainder capacity is 610.9, 669.0 and 717.7 mAh g^−1^, respectively. The CCNEs based on the grid and net deliver higher reversible discharge capacities and coulombic efficiency than those of the foil. This difference is due to the larger specific surface area and more abundant transfer paths in the grid and net substrates, which provide extra space for diffusion of the electrolyte and accommodation of the strain induced by the volume change during the electrochemical reaction, thus leading to higher charge transfer efficiency and better structural stability^41^. Figure 6c shows the rate cycling performance of these cells. The capacity and rate performance are also gradually improved as the specific surface area increases. The net sample shows the highest capacity, 589.6 mAh g^−1^, at the current density of 2500, mA g^−1^. These results indicate that the morphologies of the nanosubstrates have a significant effect on the cell performance.

**Figure 7:**
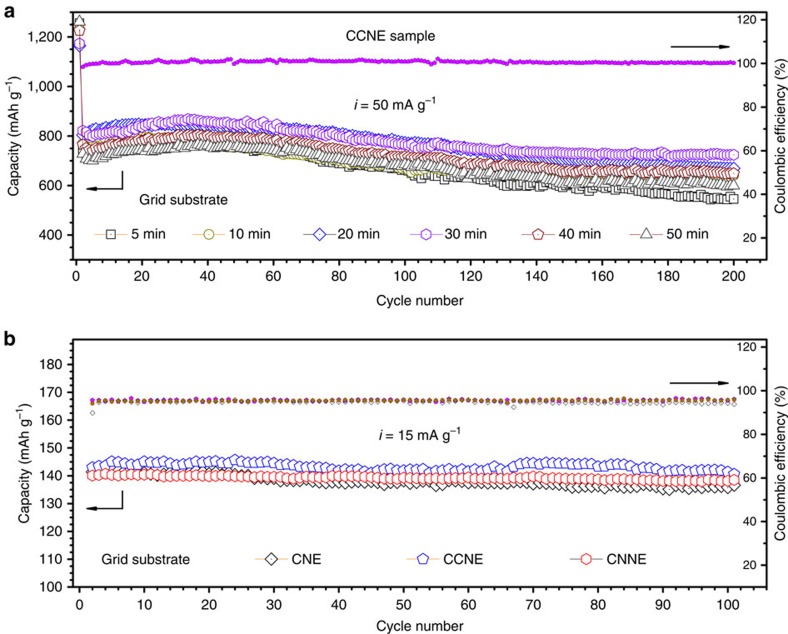
Electrochemical characterization of nanocomposite electrodes. (**a**) Cycle performance for CCNEs based on the grid substrate in half-cells with different deposition times. (**b**) Cycle performance of the full cells containing nanocomposite anodes based on the grid substrate.

We also analysed the effect of the deposited thickness of the CN_*x*_ shell on cell performance. Figure 7a shows the cycle performance of all CCNEs with different deposition times from 5 to 50 min. The reversible capacity gradually improves as the deposition time increases until 30 min. However, the capacity slightly decreases when the time increases to 40−50 min, which reveals an optimal thickness of the CN_*x*_ shell depositing on the CuO core. The longer time introduces a thicker CN_*x*_ shell, which retards the infiltration of the electrolyte into the CuO core, resulting in the decreasing charge transfer efficiency. Note that the cycle performance is improved as the deposition time increases, which indicates that the thicker CN_*x*_ shell brings better structural stability.

Full cells with the commercial LiCoO_2_ as the cathode and electrochemically activated nanocomposites (CNE, CCNE or CNNE) as the anode are fabricated to evaluate the battery performance. Figure 7b shows the cycling performance of these coin-type full cells. The working voltage of the full cells ranges from 2.4 to 3.6 V, and the current density is 15 mA g^−1^. Based on the grid substrates, the CNE/LiCoO_2_ cell delivers an initial discharge capacity of 141 mAh g^−1^ (the reversible capacity of LiCoO_2_ is 155 mAh g^−1^ when it is cycled between 3 and 4.4 V versus Li metal). The capacity fades to 136 mAh g^−1^ after 100 cycles, with 96.4% capacity retention. Comparatively, the CCNE/LiCoO_2_ and CNNE/LiCoO_2_ cells show slightly increased reversible capacities and more stable cycle performance. However, there is no obvious difference among the capacities of the full cells. This result is due to the cathode-limited design, in which the cell utilizes under stable conditions only a fraction of the capacity offered by the anode, with the cell capacity balance leading to optimal cell behaviour^42^,^43^.

### Mechanism analysis

**Figure 8:**
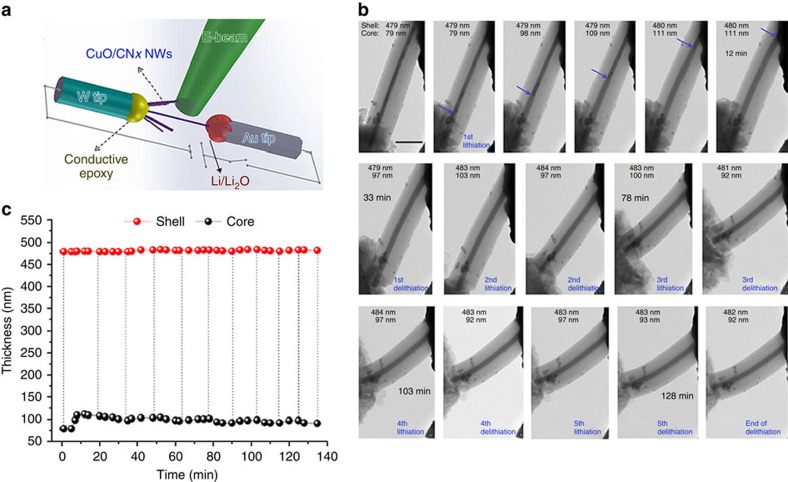
*In situ* TEM characterization of the CCNE nanocable during lithiation and delithiation. (**a**) Schematic of the *in situ* TEM device. (**b**) Time-lapse TEM images of single CCNE nanocable during lithiation and delithiation. (**c**) Thickness variation curves of the CCNE nanocable during lithiation−delithiation cycles. Scale bar in **b**, 400 nm.

The above results demonstrate that the electrode structural design of 3D arrays with core–shell nano-architecture can achieve stable performance in metal oxide anodes. Such a design has multiple advantages: First, the nitrogen-incorporated carbon framework functions as an electrical highway and a Li^+^ absorbent, so that the CuO/CN_*x*_ nanocables are electrochemically active. Figure 4j illustrates the micro-mechanism models of the nanocable, where the wire-like CuO core is a bicrystal divided by a (111) twin plane in the direction parallel to the longitudinal axis. Density functional theory calculations (Supplementary Fig. 5) display surface atomic configurations in (001), (100), (110), (111) and (−111) planes of the CuO unit cell, where the (111) and (−111) planes contain more Cu^2+^ than other surfaces^44^. According to the positive linear relationship between the electrochemical performance of CuO and the redox reaction of Cu^2+^/Cu^0^, the CuO nanowires with more exposed (111) twin planes induce more and faster Cu^2+^/Cu^0^ redox reaction, bringing higher reversible capacity and better rate capability. The (111) twin planes are also much more stable than other planes^35^, thus exhibiting better cycle performance. Moreover, the microporous CN_*x*_ shell with high nitrogen contents induces numerous pyridine- and pyrrole-like defects, adds Li insertion sites and it thus enhances Li storage capacity. Second, core–shell nano-architecture prevents fracture, where the CuO core is stabilized mechanically by the CN_*x*_ shell. The elastic CN_*x*_ layers are also believed to stabilize the electrode/electrolyte interface and thus effectively enhance the cycle stability and high rate capability^38^,^39^,^45^. This theory has been confirmed by *in situ* TEM measurements (Fig. 8, Supplementary Figs 6 and 7, and Supplementary Movie 1) of CCNE nanocables during lithiation and delithiation. Figure 8a illustrates the nanobattery for *in situ* TEM observation. Figure 8b shows a single CCNE nanocable lithiated and delithiated five times. During the initial lithiation, snapshots show a progressive lithiation mainly along the axial direction of the nanocable, where the lithiation of CuO nanowire proceeds both axially and radially, but the whole volume of the nanocable shows no evident expansion due to the mechanical buffering effect of microporous CN_*x*_ shell. Figure 8c shows the thickness variation of the nanocable during lithiation−delithiation cycles. The CuO core has an evident volume expansion by ∼93% during initial lithiation, but then its dimensions appear to stabilize during the next few cycles. Note that the thickness of the whole shell has no evident change during the cycles, indicating a good structural integrity of the nanocable electrode during electrochemical reactions. Third, 3D arrays with larger surface areas, better permeability and more active sites provide ideal conditions for the facile diffusion of the electrolyte and accommodation of the volumetric strain during electrochemical reactions, thus leading to higher charge transfer efficiency. The nanocable arrays directly grown on current collectors have good adhesion and electrical contact, reduce the path length of Li^+^ ions and electron transfer and enhance the rate capability. Fourth, the monolithic sandwich structure ensures a stable structural integrity, which prevents the electrodes from pulverization and fragmentation. It does not contain additional conductive agents or binders, and this condition improves the energy and power densities of the whole electrode. This structure also enhances the electrical contact with the current collector when it is assembled into cells. In summary, this hierarchical 3D core–shell nanostructure guarantees excellent structural properties and superior electrochemical performance.

## Discussion

Since the pioneering work of metal oxides anodes for lithium ion batteries by Tarascon in 2001 (ref. 16), more conversion materials including oxides, nitrides, fluorides and sulfides have been developed as anodes. Similar to alloy anodes, conversion anodes often suffer from low initial efficiency and severe capacity fading, originating from causes such as unstable SEI layers, material structural degradation and large volume change. Here we design a specific large-scale hierarchical structured electrode assembly, a freestanding 3D array of core–shell nanostructures, to tackle these issues. Based on above material characterizations and electrochemical measurements, the 3D CuO/CN_*x*_ core–shell nano-architecture combines features that are simultaneously attractive for electronic, mechanical and electrochemical properties necessary for stable cycling and high coulombic efficiency. The CN_*x*_ shell encapsulates the CuO electrode, limiting most SEI formation to the outer surface instead of directly on CuO active materials; at the same time, the core–shell nanostructure helps reversible accommodation of the volume change of the CuO active material. Similar approaches have also been reported in CuO@graphene and Si@C composites^46^,^47^,^48^. In the full-cell design, both cathode-limited capacity design and electrochemical prelithiation technique for the nanocomposite anodes are used to address the impact of initial capacity loss in the anode. These techniques have been well investigated and are now widely used, particularly for cells employing alloy and conversion anode materials^42^,^43^. The electrode structures developed here thus show promise for use in advanced lithium-ion batteries, and also have broader potential to be adopted for flexible energy storage devices.

In conclusion, we have developed battery electrodes combining a flexible design and 3D hierarchical nanostructures. The specific combination of composition and conformation addresses stability challenges common to many conversion electrodes and may be generalized to related systems. In addition, the simple fabrication process involving mature sputtering technology boosts prospects for scaleable production.

## Methods

### Synthesis of 3D CuO nanosubstrates

Commercially available copper foils (99.9% purity, 0.1 mm thick), copper grids (99.9% purity, 600 mesh) and copper nets (99.9% purity, 600 mesh) were used as the starting substrates to synthesize 3D CuO nanosubstrates through a vapor-phase approach^32^. In a typical procedure, copper substrates were cleaned in an aqueous 1.0 M HCl solution for 10 min, followed by repeated rinsing with distilled water. After they had been dried under a N_2_ gas flow, they were immediately heated to 600 °C for 5 h under an oxygen atmosphere in a tubular electrical furnace. In addition, CuO nanowires of similar quality were grown on the surfaces of all these copper substrates.

### Synthesis of 3D CuO/CN_*x*_ nanocomposites

A nanosized CN_*x*_ film was deposited onto CuO nanosubstrates by radio-frequency magnetron sputtering using a graphite target at 80 W and 1.0 Pa in a N_2_ atmosphere. The chamber was evacuated to a base pressure of 1.0 × 10^−5^ Pa to guarantee a clean sputtering condition. The target was pre-sputtered for 15 min to eliminate impurities. The deposition time was set in a stepwise manner to 5, 10, 20, 30, 40, 50 and 60 min.

### Synthesis of 3D Cu/CN_*x*_ nanocomposites

The obtained CuO/CN_*x*_ nanocomposites were heat treated at 300 °C for 2 h under a H_2_/Ar (10% H_2_ in volume) atmosphere at flow rate of 100 ml min^−1^ in a tubular electrical furnace.

### Characterization

The s.e.m. investigations were performed on a FEI QUANTA-250 operated at an acceleration voltage of 15 kV. The TEM observations were conducted with a JEOL-2010 microscope operated at 200 kV and a JEM-ARM200F microscope equipped with an EDX energy-dispersive spectrometer. The XRD patterns were recorded using a Rigaku Ultima IV-185 X-ray diffractometer (Cu-K*a* radiation, *λ*=1.5406 Å, 40 kV and 40 mA) at a scanning rate of 0.2^o^ s^−1^. The FT-IR spectra were obtained on a Nicolet 6700 infrared spectrometer. Raman spectra were recorded on a Labram HR 800 Raman microscope. The XPS spectra were acquired on a PHI Quantera. The *in situ* electrochemical lithiation−delithiation observations were carried out inside JEOL 3010 TEM operated at 300 KeV.

### Electrochemistry

The battery performance was evaluated by galvanostatic cycling of 2025 coin-type cells. The half-cell was made up of nanocomposites as the working electrode and lithium foil as the counter electrode. The full cell was composed of commercial LiCoO_2_ as the cathode and nanocomposites as the anode. The electrolyte was 1.0 M LiPF_6_ in 1:1 ratio by volume of ethylene carbonate/dimethyl carbonate. A Celgard 2400 membrane was employed as the separator. Before assembling the full cells, nanocomposite anodes were electrochemically activated for three cycles in half-cells to eliminate the capacity loss in the first cycle. Being a cathode-limited design, the cell balance value (the capacity ratio of the anode to the cathode) is designed to be >1 to ensure efficient utilization of the cathode materials. The cells were assembled in an Ar-filled glove box, then aged for 24 h before the electrochemical tests. Cyclic voltammetry and electrochemical impedance spectroscopy measurements were performed on a ZAHNER PP211 electrochemical workstation. Galvanostatic charge–discharge tests were carried out with a Land CT2001A battery tester. The weight of the CuO active materials was determined by subtracting the weight of the residual Cu substrate from the overall weight of the CNE, by using a hydrochloric acid corrosion method. The weight of the CN_*x*_ coating layer was determined by subtracting the weight of the CNE from the overall weight of CCNE. Supplementary Table 1 illustrates the areal masses of all the nanocomposite electrodes. For the CCNEs, the specific capacity was calculated based on the total mass of CuO/CN_*x*_ active materials. All of the weight values were obtained with a high-precision BT 25S electronic balance.

## Additional information

**How to cite this article:** Tan, G. *et al*. Freestanding three-dimensional core–shell nanoarrays for lithium-ion battery anodes. *Nat. Commun.* 7:11774 doi: 10.1038/ncomms11774 (2016).

## Supplementary Material

Supplementary Information Supplementary Figures 1-7, Supplementary Table 1, Supplementary Notes 1-2 and Supplementary References

Supplementary Movie 1 In-situ TEM time-lapse movie of a single-sided coating CuO/CN_x_ nanocable during the first lithiation process. Movie shows progressive lithiation mainly along the axial direction of the nanocable and morphological stability during lithiation. Curvature of nanocable is a result of the stable CN_x_ shell resisting the large volume expansion of CuO core.

## Figures and Tables

**Figure 1:**
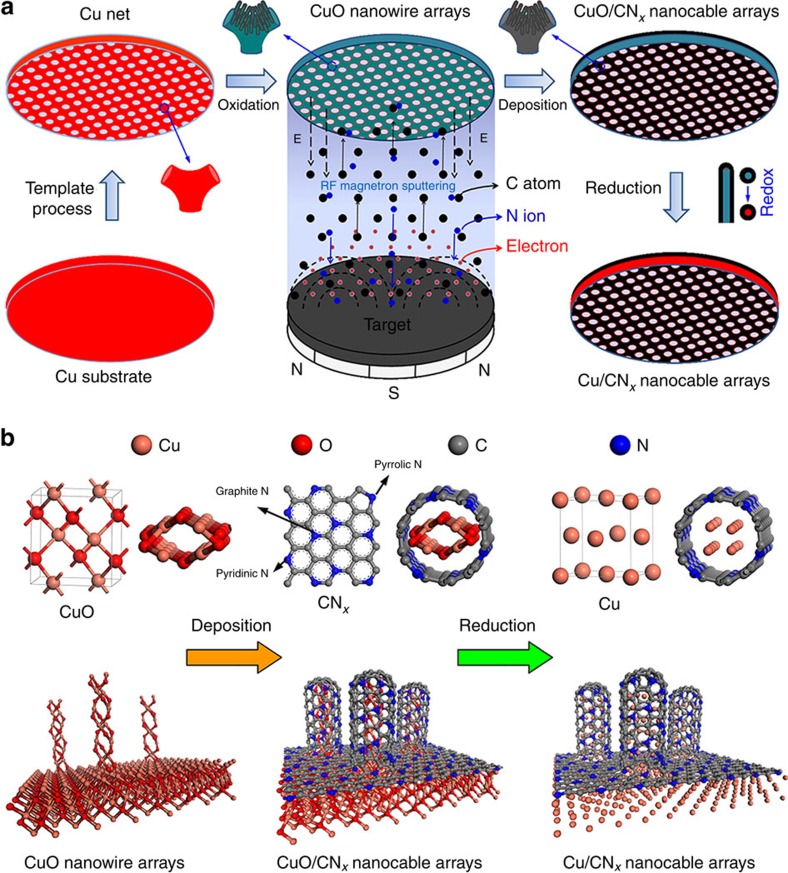
Schematic of 3D arrays with the core–shell nano-architectural design. (**a**) The typical two-step electrode design consisting of the oxide growth of CuO nanowires onto a copper net substrate followed by radio-frequency magnetrson sputtering of CN_*x*_ films, forming a binder-free 3D array with core–shell nano-architecture. (**b**) Theoretical structural modelling showing the micro-mechanism of CNE, CCNE and CNNE formation.
