# Supplementary material for: Freestanding three-dimensional core–shell nanoarrays for lithium-ion battery anodes
Source: Nat Commun. 2016 Jun 3;7:11774. doi: 10.1038/ncomms11774 (PMC4895809; doi:10.1038/ncomms11774)
Supplement: Supplementary Information — Supplementary Figures 1-7, Supplementary Table 1, Supplementary Notes 1-2 and Supplementary References [file ncomms11774-s1.pdf]

Supplementary Figure 1

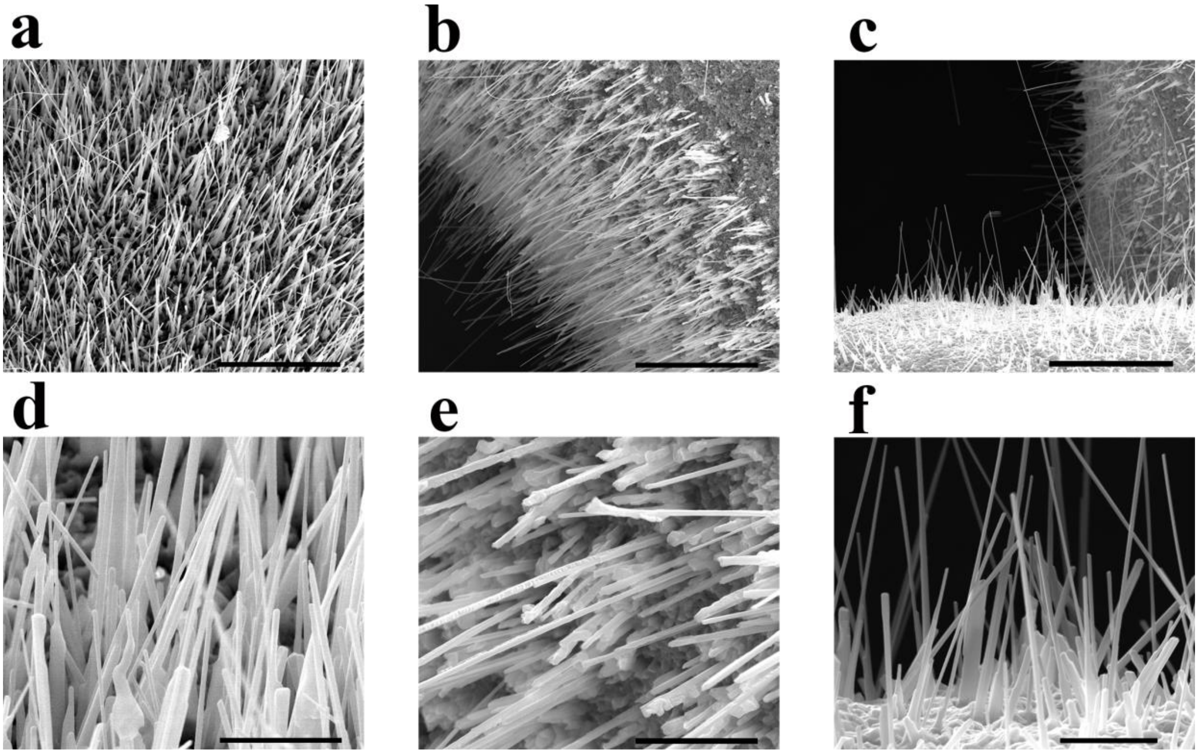

Additional SEM images: SEM micrographs of CuO nanowires grown on the foil (a,d), net (b,e), and grid (c,f) substrates. (scale bars in a−c, 10 um; scale bars in d−f, 2 um)

Supplementary Figure 2

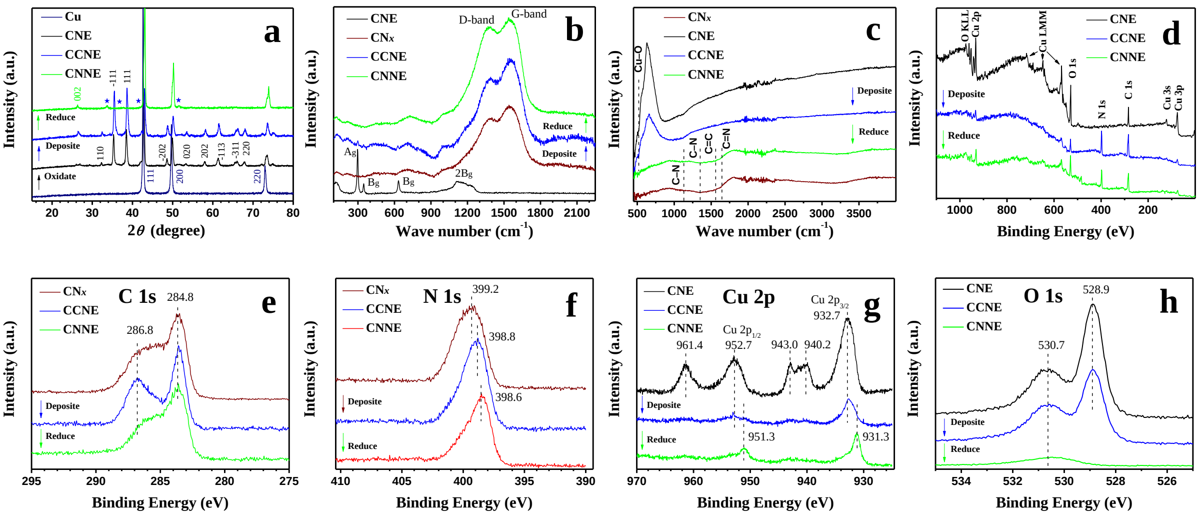

Structure and chemical composition of nanocomposite electrodes: (a) XRD, (b) Raman, (c) FT-IR, and (d) XPS spectra of the nanosubstrates, and (e–h) High-resolution XPS spectra of C1s (e), N1s (f), Cu2p (g), and O1s (h). (In these and subsequent figures, CNE = CuO nanowires electrode, CCNE = CuO/CN_x nanocables electrode, and CNNE = Cu/CN_x nanocables electrode)

Supplementary Figure 3

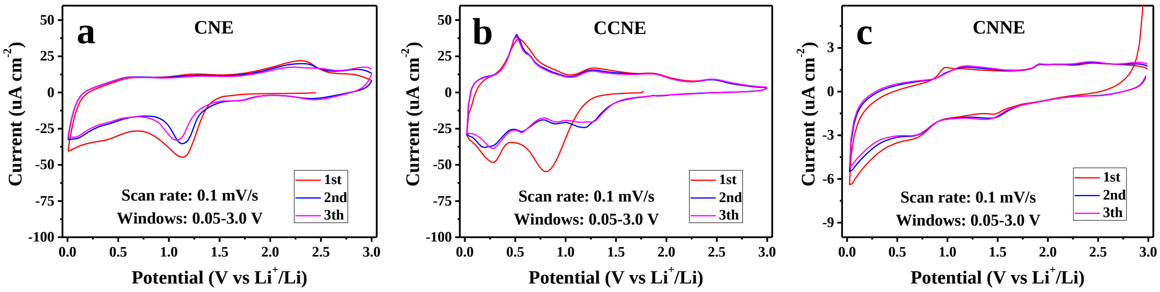

Cyclic voltammetry tests: Cyclic voltammograms of the (a) CNE, (b) CCNE, and (c) CNNE samples.

Supplementary Figure 4

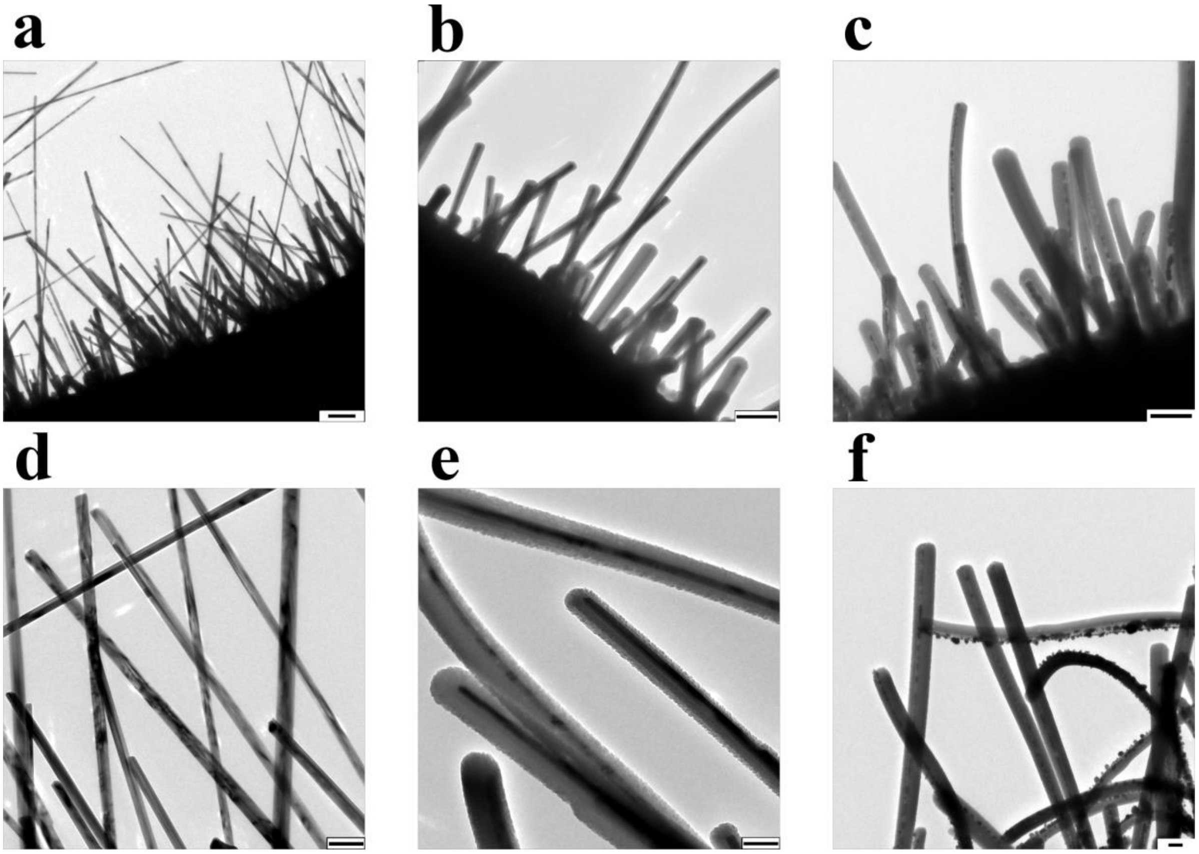

Additional TEM images: TEM micrographs of the CNE (a,d), CCNE (b,e), and CNNE (c,f) based on copper grid substrates. The under TEM images represent the top of long nanowires for the CNE (d), CCNE (e), and CNNE (f). (scale bars in a−c, 600 nm; scale bars in d−f, 200 nm)

Supplementary Figure 5

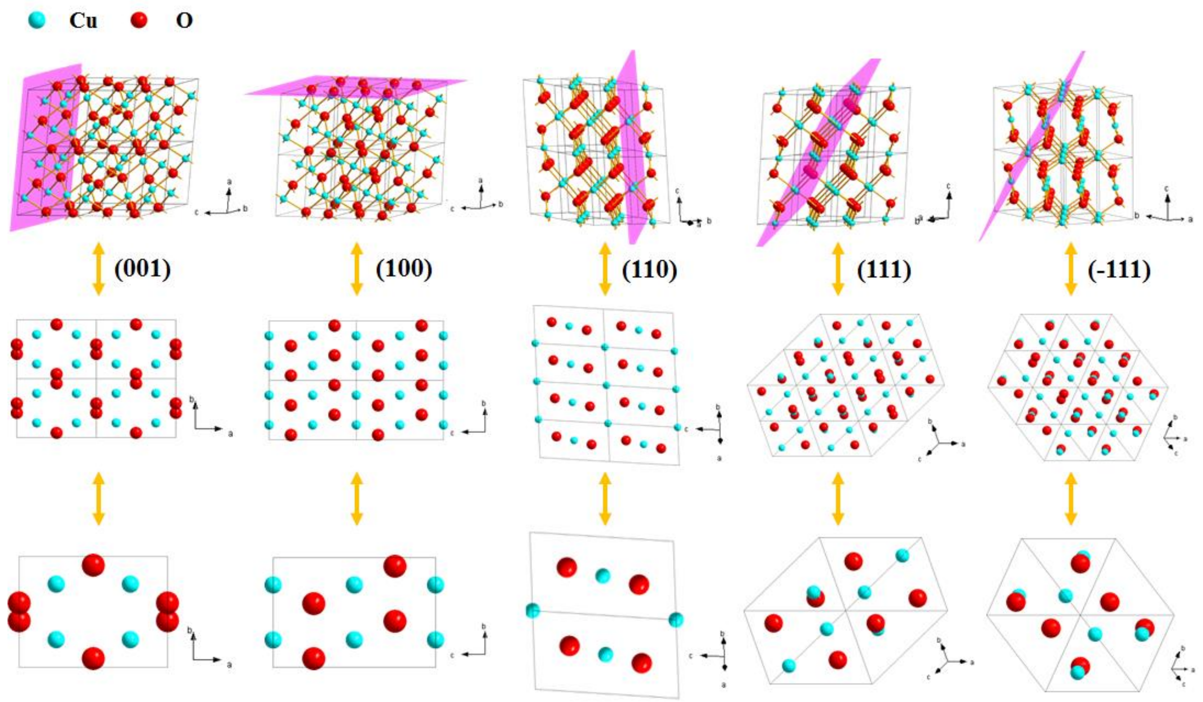

DFT models of different planes of CuO: The 3D and 2D surface atomic configurations in the (001), (100), (110), (111), and (−111) planes of CuO.

Supplementary Figure 6

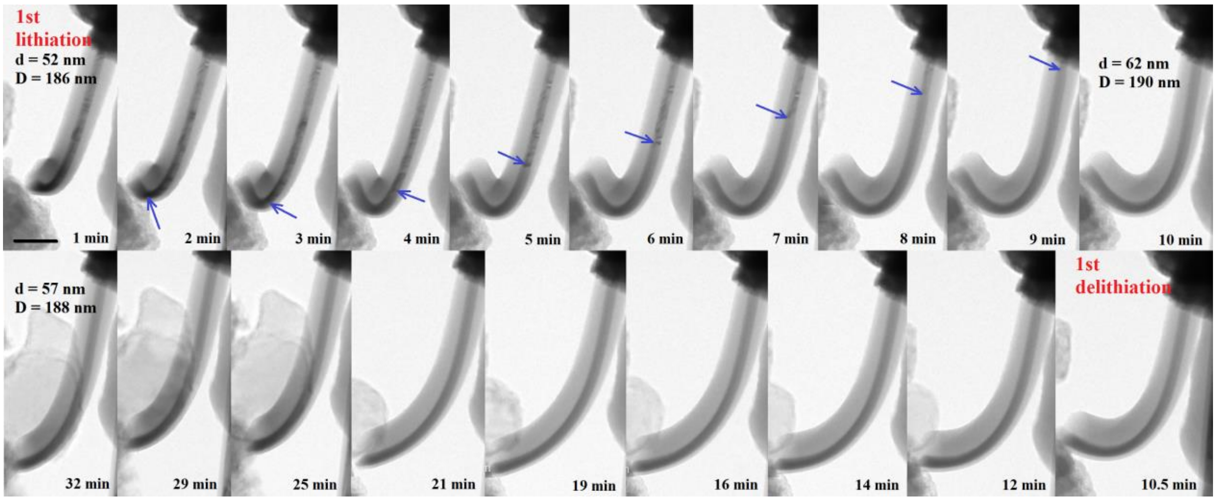

Additional *In-situ* TEM images: *In-situ* TEM time-lapse images of the CCNE nanocable during the first lithiation and delithiation. (d = the thickness of CuO core nanowire; D = the thickness of the whole CuO/CN_x nanocable; scale bar, 200 nm)

Supplementary Figure 7

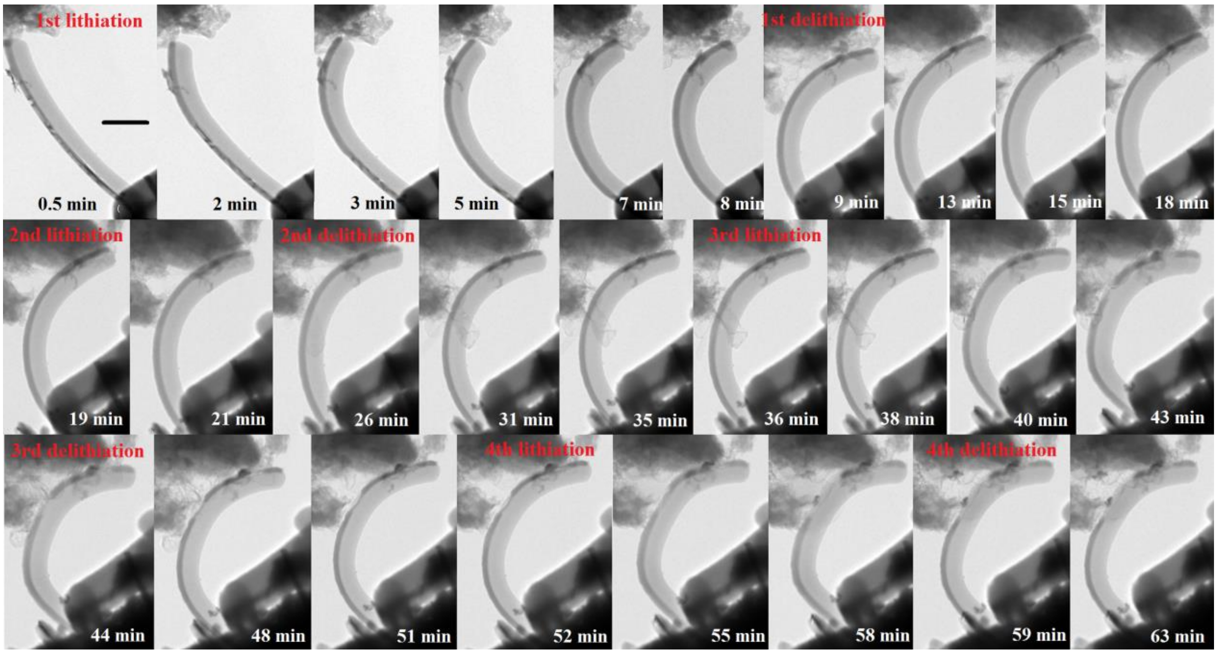

Additional *In-situ* TEM images: *In-situ* TEM time-lapse images of a single-sided coating CCNE nanocable during lithiation and delithiation cycles. The curvature formation during the first lithiation is due to the stable CN_x shell that can resist huge volume expansion of CuO nanowire. A fracture forms at 43 min, and the nanowire can still be reversible (de)lithiated after the fracture formation ─ importance of the CN_x shell. (scale bar, 200 nm)

Supplementary Table 1

The areal masses of all the nanocomposite electrodes[α]

Sample	Monolithic electrode				CuO active material	CN_x active material	$CuO@CN_x$ loading percent
	Cu	CNE	CCNE	CNNE			
Foil	22.56	36.16	36.43	34.25	13.60	0.27	38.07%
Grid	10.10	21.40	21.60	19.83	11.30	0.20	53.24%
Net	7.94	17.04	17.25	15.80	9.10	0.21	53.97%

[α] The areal mass of each nanocomposite sample was obtained by calculating the average value of five electrode samples; The residual Cu substrate was prepared by removing CuO from the CNE sample using a hydrochloric acid corrosion method; The CCNE sample was prepared by depositing CN_x layer onto both two sides of the CNE sample, the deposition time for each side was 30 min. (unit: mg/cm^2) (In this table, CNE = CuO nanowires electrode, CCNE = CuO/CN_x nanocables electrode, and CNNE = Cu/CN_x nanocables electrode)

Supplementary Note 1

The structure and chemical composition of all samples were characterized by XRD, Raman, FT-IR, and XPS, as shown in Supplementary Figure 2. In Supplementary Figure 2a, XRD patterns reveal typical variations of diffraction peaks in the course of copper oxidation, CN_x deposition, and CuO reduction. The XRD reflection of the copper substrate shows three typical sharp diffraction peaks, located at 43.2^o (111), 49.5^o (200), and 73.9^o (220), which can be indexed to the face-centered cubic structure[1,2]. In the CNE, other than those from copper diffractions, the rest of the peaks can be indexed to a monoclinic phase for CuO nanowires[1,2]. Comparatively, all diffraction peaks of the CCNE are almost in agreement with those of the CNE, except for a weak peak at 26.2^o corresponding to the (002) plane of graphite-like carbon nitrides. After the reduction, the XRD reflection of the CNNE almost recovers the diffraction feature of the metallic copper. Nevertheless, some slight diffraction peaks, identified by blue stars, are observed in all nanosubstrates, owing to a spot of Cu_2O[1].

In Supplementary Figure 2b, three characteristic bands at 292, 341, and 626 cm^{-1} are observed in the Raman spectrum of the CNE, corresponding to one A_g and two B_g modes of vibrations of CuO[2,3]. The other broad band at about 1100 cm^{-1} corresponds to the $2B_g$ modes of vibrations of CuO[3]. These results indicate the single-phase property and high crystallinity of CuO nanowire arrays. In the as-deposited CN_x film, two intense bands at 1355 and 1593 cm^{-1} are attributed to the D-band and G-band of graphite-like carbon nitrides, respectively[4]. Comparatively, the CCNE shows an overlapped feature of both CuO and CN_x, indicating that the CuO and CN_x are coexisting in the CCNE. The CNNE shows a similar Raman spectrum to that of the as-deposited CN_x film. It indicates that the CuO cores in the CCNE are reduced into the metallic copper.

In Supplementary Figure 2c, the FT-IR spectroscopy analysis provided further evidence for the oxidation of Cu^{2+} in CuO and the chemical structure of the CN_x film. In the CNE, bands at about 500 cm^{-1} are associated with Cu−O stretching modes in monoclinic CuO phase[5,6]. Note that the IR spectrum of CuO shows a prominent feature at wave numbers above 700 cm^{-1}, that is, the transmittance is small above 700 cm^{-1}, and this is due to the scatterings of light through a highly crystalline CuO film of optically anisotropic monoclinic structure[5]. Similar spectral features are also observed for the CCNE, but with a depressed intensity due to the coating effect of the CN_x film. In the as-deposited CN_x film and reductive CNNE, both of the spectra show a band at ~1575 cm^{-1} from the C=C vibrations in the graphitic layers and

a band at ~1646 cm^{-1} from the C=N bonds. The bands at around 1140, 1280, and 1350 cm^{-1} are attributed to the tetrahedral C−N bonds[4,7].

Supplementary Figure 2d shows a set of parallel XPS survey scans for comparison. The XPS spectra of all composites demonstrate the presence of Cu, O, and C elements. The C1s peaks were corrected to be 284.8 eV, and all the other peaks were corrected accordingly. An obvious peak located at 398.0 eV in the CCNE and CNNE, but not the CNE, is attributed to the nitrogen-substituted carbon during RF sputtering. In Supplementary Figure 2e, the C1s peak showing an asymmetry in the left part at about 286.8 eV can be attributed to C−N bonds, and the main peak at 284.8 eV is related to graphite-like sp^2 C, which indicates that most of the C atoms in the CN_x are arranged in a conjugated honeycomb lattice[4,8]. It is found that the main peak shifts slightly to higher binding energy compared to that in the graphitized carbon materials, suggesting the combination between C and N[9]. In Supplementary Figure 2f, the N1s peak also shows an asymmetry. The major component at about 398.7 eV, due to the pyridinic nitrogen, and shoulder peaks at 400.1−401.2 eV are assigned to the pyrrolic nitrogen in the terminal C−N groups and quaternary nitrogen in the graphite C−N bonds[4,10]. This finding indicates that the nitrogen-substituted carbon creates more pyridine- and pyrrole-like defects in the carbon framework and generates more active sites for the Li storage. In Supplementary Figure 2g, the Cu2p spectra of the CNE present two main peaks of Cu $2p_{3/2}$ and Cu $2p_{1/2}$. The fit of the $Cu2p_{3/2}$ peaks reveals a main peak at 932.7 eV, and two characteristic satellite peaks at 940.2 and 943.0 eV. The peak-fit of $Cu2p_{1/2}$ spectra gives a main peak at 952.7 eV, accompanied by a satellite peak at 961.4 eV. These features are typical of Cu^{2+} species and confirm that the grown nanowires are CuO[11]. The difference in binding energy between $Cu2p_{3/2}$ and $Cu2p_{1/2}$ is 20.0 eV, which is also in good agreement with the +2 copper oxidation state[12,13]. In the CCNE, due to the coating effect of the CN_x film, the intensity of the Cu2p peaks is obviously decreased. The main peaks show scarcely any shifts, indicating that the dominant valence of Cu is divalent. Comparatively, the main peaks of $Cu2p_{3/2}$ and $Cu2p_{1/2}$ are shifted to the lower value by 1.4 eV in the CNNE. These peaks are typical of Cu^0 species and consistent with those observed in metallic copper[14]. In Supplementary Figure 2h, the O1s spectra of the CNE and CCNE exhibits a primary peak at 528.9 eV and a satellite peak at 530.7 eV, which are assigned to the lattice oxygen of CuO and chemisorbed oxygen[15], respectively. In the CNNE, however, the lattice oxygen signal has disappeared, and the chemisorbed oxygen is also inconspicuous, indicating the full reduction of CuO into metallic copper.

Supplementary Note 2

Supplementary Figure 3 shows the cyclic voltammograms of all samples during the first three cycles. In Supplementary Figure 3a, the CNE sample exhibits two cathodic peaks at the potential of 1.60─1.80 V and 0.70─1.50 V in all three cycles, which correspond to the reduction from CuO to intermediate composite copper oxide phase, to Cu_2O and further decomposition into Cu and Li_2O[2,13,16]. Another weak peak near 0.5 V is attributed to the reductive decomposition of the electrolyte and subsequent formation of solid-electrolyte interface (SEI) layer between CNE and electrolyte[4]. During the anodic scanning, there is a board peak located at 1.9─2.4 V, and another small peak at 2.5─2.7 V, which can be ascribed to the formation of Cu_2O and the oxidation of Cu_2O to CuO[13]. By contrast, in Supplementary Figure 3b, the CCNE sample shows a strong and broad peak located at 0.75 V and another peak at 0.25 V during the first cathodic polarization process. The first peak at 0.75 V is mainly ascribed to the stable formation of SEI layer, it almost disappears in the subsequent cycles, indicating that the SEI layer is mainly formed during the first cycle, due to the minimized volume expansion of core-shell nanocables. The second cathodic peak at 0.25 V corresponds to the lithiation of the CN_x shell. In the following half cycles, an obvious anodic peak located at 0.50 V, representing the delithiation of the CN_x shell. After the reduction, in Supplementary Figure 3c, the CNNE sample shows a similar cyclic voltammetry characteristic to that of the carbon nitrides compounds[4,17,18].

Supplementary References

1. Cao, M., Hu, C., Wang, Y., Guo, Y., Guo, C. & Wanga, E. A controllable synthetic route to Cu, Cu_2O, and CuO nanotubes and nanorods. *Chem. Commun*. 1884−1885 (2003).

2. Zhang, Q., Xu, D., Zhou, X., Wu, X. & Zhang, K. In situ synthesis of CuO and Cu nanostructures with promising electrochemical and wettability properties. *Small* **10**, 935−943 (2014).

3. Zhu, J., Zeng, G., Nie, F., Xu, X., Chen, S., Han Q. & Wang, X. Decorating graphene oxide with CuO nanoparticles in a water–isopropanol system. *Nanoscale* **2**, 988−994 (2010).

4. Mao, Y., Duan, H., Xu, B., Zhang, L., Hu, Y., Zhao, C., Wang, Z., Chen, L. & Yang, Y. Lithium storage in nitrogen-rich mesoporous carbon materials. *Energy Environ. Sci*. **5**, 7950−7955 (2012).

5. Maruyama, T. Copper oxide thin films prepared by chemical vapor deposition from copper dipivaloylmethanate. *Sol. Energ. Mat. Sol. C*. **56**, 85−92 (1998).

6. Xu, Y., Chen, D. & Jiao, X. Fabrication of CuO pricky microspheres with tunable size by a simple solution route. *J. Phys. Chem. B* **109**, 13561−13566 (2005).

7. Veith, G. M., Baggetto, L., Adamczyk, L. A., Guo, B., Brown, S. S., Sun, X.-G., Albert, A. A., Humble, J. R., Barnes, C. E., Bojdys, M. J., Dai, S. & Dudney, N. J. Electrochemical and solid-state lithiation of graphitic C_3N_4. *Chem. Mater*. **25**, 503−508 (2013).

8. Wang, H., Zhang, C., Liu, Z., Wang, L., Han, P., Xu, H., Zhang, K., Dong, S., Yao, J. & Cui, G. Nitrogen-doped graphene nanosheets with excellent lithium storage properties. *J. Mater. Chem*. **21**, 5430−5434 (2011).

9. Wu, C., Zhu, X., Wang, C., Sheng, H., Yang, J. & Xie, Y. Bamboolike carbon nitride nanotubes ($C_9N_5H_3$): Atomic-scale construction, synthesis and lithium battery applications. *Appl. Phys. Lett*. **90**, 113116 (2007).

10. Qie, L., Chen, W., Wang, Z., Shao, Q., Li, X., Yuan, L., Hu, X., Zhang, W. & Huang, Y. Nitrogen-doped porous carbon nanofiber webs as anodes for lithium ion batteries with a superhigh capacity and rate capability. *Adv. Mater*. **24**, 2047−2050 (2012).

11. Zheng, S., Hu, J., Zhong, L., Song, W., Wan, L. & Guo, Y. Introducing dual functional CNT networks into CuO nanomicrospheres toward superior electrode materials for lithium-ion batteries. *Chem. Mater*. **20**, 3617−3622 (2008).

12. Akhavan, O., Azimirad, R., Safad S. & Hasani, E. CuO/Cu$(OH)_2$ hierarchical nanostructures as bactericidal photocatalysts. *J. Mater. Chem*. **21**, 9634−9640 (2011).

13. Wang, C., Li, Q., Wang, F., Xia, G., Liu, R., Li, D., Li, N., Spendelow, J. S. & Wu, G. Morphology-dependent performance of CuO anodes via facile and controllable synthesis for lithium-ion batteries. *ACS Appl. Mater. Interfaces* **6**, 1243−1250 (2014).

14. Gelves, G. A., Murakami, Z. T. M., Krantz, M. J. & Haber, J. A. Multigram synthesis of copper nanowires using ac electrodeposition into porous aluminum oxide templates. *J. Mater. Chem.* **16**, 3075−3083 (2006).

15. Zhang, H., Cao, J.-L., Shao G-S. & Yuan, Z.-Y. Synthesis of transition metal oxide nanoparticles with ultrahigh oxygen adsorption capacity and efficient catalytic oxidation performance. *J. Mater. Chem.* **19**, 6097−6099 (2009).

16. Reddy, M. V., Yu, C., Fan, J., Loh, K. P. & Chowdari, B. V. R. Li-cycling properties of molten salt method prepared nano/submicrometer and micrometer-sized CuO for lithium batteries. *ACS Appl. Mater. Interfaces* **5**, 4361−4366 (2013)

17. Li, Z., Xu, Z., Tan, X., Wang, H., Holt, C. M. B., Stephenson, T., Olsen, B. C. & Mitlin, D. Mesoporous nitrogen-rich carbons derived from protein for ultra-high capacity battery anodes and supercapacitors. *Energy Environ. Sci.* **6**, 871−878 (2013).

18. Belen Jorge, A., Cora, F., Sella, A., McMillan, P. F. & Brett, D. J. L. Electrochemical properties of graphitic carbon nitrides. *Int. J. Nanotechnol.* **11**, 737−746 (2014).
